# E-scooter attitudes and risk-taking behaviours: an international systematic literature review and survey responses in the West Midlands, United Kingdom

**DOI:** 10.3389/fpubh.2023.1277378

**Published:** 2023-10-13

**Authors:** Nathalie Burt, Zubair Ahmed

**Affiliations:** ^1^Institute of Inflammation and Ageing, University of Birmingham, Birmingham, United Kingdom; ^2^Birmingham Medical School, University of Birmingham, Birmingham, United Kingdom; ^3^Centre for Trauma Sciences Research, University of Birmingham, Birmingham, United Kingdom; ^4^NIHR Surgical Reconstruction and Microbiology Research Centre, Queen Elizabeth Hospital, Birmingham, United Kingdom

**Keywords:** electric scooter, risk-taking behaviour, survey, systematic review, trauma

## Abstract

**Introduction:**

Micromobility initiatives, including electric scooters (e-scooters), are part of the United Kingdom government’s sustainability drive. Since summer 2020, multiple trials have been conducted across the United Kingdom. Safety concerns have been raised around e-scooters joining other vehicles on United Kingdom roads, alongside the numerous private e-scooters illegally ridden on public land. Although literature has been published abroad on perceptions, risk-taking behaviours and attitudes surrounding e-scooters, independent United Kingdom research has concentrated on analysing trauma. Our aim was to identify common themes and recommendations to form conclusions on factors affecting e-scooter trauma hospital admissions.

**Methods:**

A systematic literature search in June 2023 extracted studies focused on the primary outcomes of risk factors, perceptions, and attitudes surrounding e-scooters globally from the EMBASE, PubMed, and Web of Sciences databases. Two independent reviewers conducted a critical appraisal to extract potential biases and study characteristics. A critical appraisal skills programme (CASP) analysis was also completed. Two online surveys distributed in Birmingham and Wolverhampton focused on: public perception towards e-scooters, and road user attitudes around e-scooters. The target population was residents of the West Midlands who were both riders and non-riders of e-scooters. The surveys were opened in late-March 2023 and closed in late-July 2023.

**Results:**

443 studies were retrieved with 13 studies being eligible according to our inclusion and exclusion criteria. CASP assessment concluded that the studies were of good quality, however heterogeneity meant sample sizes could not be meaningfully aggregated. Many studies focused on safety concerns whilst others observed risk-taking behaviour, non-rider perceptions, and infrastructure. Our surveys received 299 responses and respondents reported risk-taking behaviours such as pavement riding, alcohol consumption, and minimal helmet use. However, positive opinions were expressed on e-scooter convenience but concerns were raised regarding rider and non-rider safety.

**Discussion:**

Whilst global literature had investigated e-scooter attitudes, risk-taking behaviours and perceptions, there was no comparable independent United Kingdom literature. Our literature review and analysis of survey responses concluded that e-scooters were perceived as a sustainable form of transport; however, safety concerns were raised. Our study points to risk-taking behaviours by riders being associated with admissions into hospital emergency departments. We conclude that well maintained infrastructure could improve the safety of both e-scooter riders and vulnerable pedestrians, whilst education and enforcement of clear rules may reduce risk-taking behaviour. The recommendations found in the PACTS reports, and documents from the RNIB confirm our findings. We recommend that hospital data and future studies should differentiate between private and rental e-scooters for robust conclusions to be made.

## 1. Introduction

Micromobility initiatives, including pedal bicycles, electric bicycles (e-bikes), and electric scooters (e-scooters), have been a part of the United Kingdom government’s sustainability drive to reduce carbon emissions (1). At the end of summer 2020, e-scooter rental (rideshare) trial schemes began across England, including the West Midlands, to determine whether e-scooters should be legalised for use on public roads (2). Since then, usage has greatly increased with an associated increase in e-scooter related injuries presenting to hospital (3–5).

The number of studies on e-scooter trauma and perceptions has significantly increased in recent years with further studies expected to be published in the latter half of 2023. Several studies have been conducted in the United Kingdom around e-scooter injury patterns and demographics (3, 6, 7), however, we have found no analysis on public attitudes and risk-taking behaviours apart from those by the Department for Transport and the micromobility companies themselves (1, 8, 9). Until the International Classification of Diseases added a separate code for standing e-scooters in October 2020 (10), there was no assurance that studies prior to this distinguished between injuries related to standing e-scooters vs. electric mobility vehicles, also commonly described as ‘e-scooters’.

Data are available on the number of rides for rental e-scooters, but no comparable data are available for their private counterparts. There is a much higher number of private e-scooters compared to rental in the United Kingdom. In 2021, the number of private e-scooters was estimated at approximately 750,000 compared to *circa* 20,000 rental e-scooters (11), which have stricter safety features. The number of private e-scooters, although illegal for use outside of private land, is likely to have increased, possibly due to perceived low law enforcement (4).

Safety concerns have been expressed by multiple interested parties, including the Royal Society for the Prevention of Accidents (ROSPA) (12), the Royal National Institute of Blind People (RNIB) (13), and the Parliamentary Advisory Council for Transport Safety (PACTS) (14). Guidance does exist around safe behaviours whilst using an e-scooter but is not always followed. Risk-taking behaviours by e-scooter riders increases the risk of greater injury and potentially contributes to negative public perception.

Researchers from other countries have investigated behaviours and attitudes of e-scooter riders and uncovered: a concern over a lack of clear legislation; a difference in safety perception between riders and non-riders; and a generally negative perception of e-scooter riders. A systematic review on psychosocial risky behaviours of e-scooters by Useche et al. (15) found that e-scooters were mostly used by young, highly educated, urban-dwelling males, usually for short trips. They highlighted that groups with low risk perception were more susceptible to undertake risky behaviours, contributing to a higher risk of being involved in a collision. The study endorsed the development and enforcement of specific e-scooter traffic laws and education as ways to reduce risky behaviours and e-scooter accidents.

Finding a balance between encouraging micromobility initiatives and their safety issues is the difficult challenge faced by authorities globally. For example, in Singapore where new rules were implemented in February 2019 to reduce the severity of e-scooter vs. pedestrian collisions (16), it seemed to de-incentivise e-scooter riders to continue using this mode of transport. Che et al. (17) used virtual reality scenarios to understand the experiences of pedestrians and e-scooter riders in a series of interactions. They concluded that pedestrians and riders felt safest in face-to-face interactions but noted that riders felt more unstable going at the slower speeds, despite an increased perception of safety amongst the pedestrian participants.

Our study aimed to provide insights into various risk-taking activities driving both traumatic injuries and understand public perceptions of e-scooters combining a systematic review of international literature and analysis of online surveys in the West Midlands, United Kingdom. The surveys aimed to capture information regarding risk taking behaviours and the general public perceptions regarding the use of e-scooters. Our study findings could inform future decision making regarding accident prevention involving e-scooters ridden on public land.

## 2. Materials and methods

### 2.1. Study design

In this non-randomised study, we completed a systematic review on the global literature available on public attitudes and risk-taking behaviours surrounding e-scooters and compared this to the results of two surveys completed in the West Midlands, England.

### 2.2. Literature search strategy

Our systematic review was not prospectively registered with PROSPERO; however, we followed the Preferred Reporting of Items for Systematic Reviews and Meta-analysis (PRISMA) guidelines (18). For the systematic review, two independent reviewers (NB and ZA) conducted the literature search. Strict inclusion and exclusion criteria were applied to enable a strong focus limited to the search topic. After removing duplicates, title screening occurred before subsequent abstract and full-text analysis. Discrepancies were resolved through discussion and eventual mutual agreement. The literature search was performed on June 7, 2023 using the term ‘e-scooter’ across three databases: EMBASE, PubMed and Web of Sciences. Due to resource limitations, we restricted ourselves to three databases, with MEDLINE and Scopus considered high quality databases but ultimately not included in the search. We used EndNote software to search simultaneously for alternative adjacent keywords, e.g., ‘electric scooter’. To obtain a comprehensive list of potential studies, no further keywords were used in the initial strategy. The primary outcomes were screened for one or more of: risk factors; perceptions; or attitudes of e-scooter usage. A secondary outcome was a comparison with bicycles. Studies were excluded if the primary outcome was e-scooter sustainability. Searches were limited to English language only. Boolean search operators were also combined with search terms to ensure the maximal number of studies were retrieved (Table 1).

**Table 1:** Boolean search criteria.

Boolean search criteria
“e-scooter” AND/OR “electric scooter”
AND “risk factors” AND/OR “risk-taking behaviours” AND/OR “perceptions” AND/OR “attitudes”
AND NOT “sustainability”

A pre-designed spreadsheet was used to extract data on study characteristics. Each study was analysed for the overall theme, subthemes, study demographics, methods and duration. The themes were mined from the literature by the two independent reviewers, discussed and finalised. Any differences of opinion or uncertainty were resolved through discussion (Table 2).

**Table 2:** Inclusion and exclusion criteria of literature search.

Inclusion criteria	Exclusion criteria
Full text analysis	Full text unavailable
Primary outcome one or more of the following: risk factors, perception, or attitudes of e-scooter usage	Primary outcome not surrounding risk factors, perception, or attitudes of e-scooter usage
Primary research	Not primary research, e.g., systematic review
Focus on e-scooter	Primary outcome focused on sustainability
Full text in English	Full text not in English

### 2.3. Study quality

The quality of each study was assessed using the Critical Appraisal Skills Programme (CASP) qualitative research checklist (19). The two reviewers (NB and ZA) completed this analysis for each study and came to a final agreed decision through discussion. The standard tools to assess risk of bias were not suitable, due to the qualitative and observational nature of the data collected. The CASP checklist was used with a quantifiable number assigned to each response. Ten domains were assessed and an overall quality for each study was calculated. The CASP checklist has three levels for each domain, to which the two reviewers assigned a score. ‘No’ scored one point, ‘Cannot tell’ scored two points, and ‘Yes’ scored three. The maximum quality score would therefore be 30 and the minimum 10. A score of 24 or more was considered of good quality.

### 2.4. Survey methods

Two questionnaires were distributed using various methods: University of Birmingham email bulletins; social media platforms such as WhatsApp groups; and physical posters with a QR code to access the questionnaires. The posters were placed in visible locations in the area surrounding the Queen Elizabeth Hospital Birmingham (QEHB) and New Cross Hospital, Wolverhampton. Local schools and railway stations were contacted as potential sites for posters, however, they declined. Respondents were anticipated to be either permanent or temporary residents in the West Midlands due to the distribution methods. The surveys were opened in late-March 2023 and closed in late-July 2023, giving a study period of 4 months.

The questionnaires investigated: public opinion of e-scooters and road user attitudes to e-scooters (Appendix 1). An opportunity for respondents to discuss personal observations and self-reported e-scooter usage was made available in both surveys. Respondents could answer either or both questionnaires. At the end of each questionnaire respondents could supply free text comments on the subject. There was no monetary or other incentive given for completion.

Ethics approval was not required due to the data collection method maintaining anonymity and none of the surveys contained questions on identifiable data. Responses were stored in an encrypted and password protected spreadsheet.

### 2.5. Statistical analysis

All of the included studies from the literature search were qualitative in nature and included surveys and observational studies. The heterogeneity of data collected between each study did not allow us to record sufficient quantitative data to perform a meta-analysis. Therefore, we narratively synthesised the main themes reported by the included studies.

## 3. Results

### 3.1. Literature review results

#### 3.1.1. Study selection

**Figure 1:**
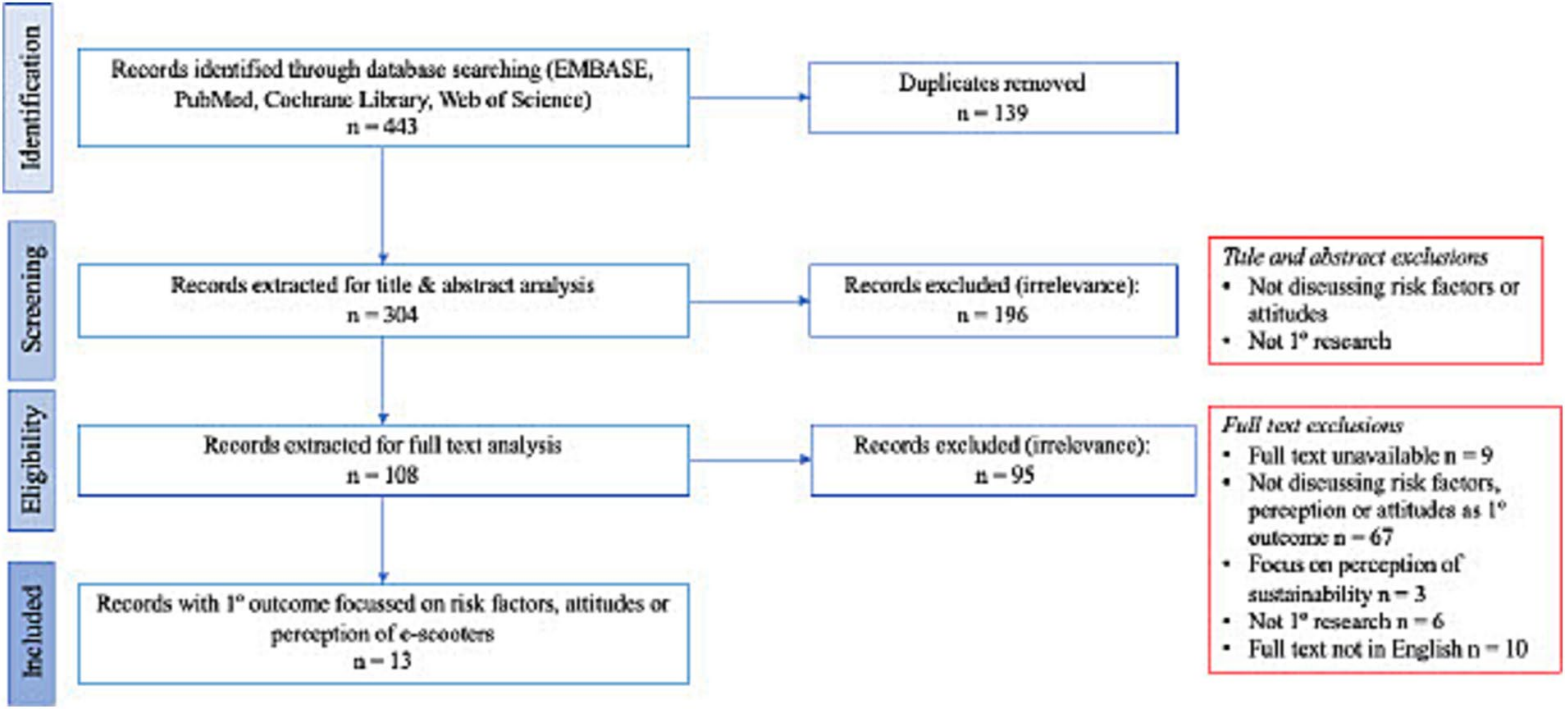
PRISMA flowchart detailing selection process.

The literature search strategy resulted in 443 studies being identified from the three databases, with 139 duplicates being removed. Abstract and title screening removed a further 196 studies as irrelevant to our main outcomes. Subsequent selection and critiquing resulted in 13 studies being subject to full-text analysis. The process is depicted in Figure 1 in the form of a PRISMA flowchart.

#### 3.1.2. Study characteristics

Multiple methods of data collection were used in the included studies. For example, seven of the 13 studies used surveys alone (20–26), one used one-on-one interviews (27), two observed behaviours (28, 29), one compiled newspaper articles (30) and two combined surveys with observations (19, 24). Gossling (25) compiled news articles from nine countries regarding the media perception of e-scooters. Uluk et al. (21) was the only study in this review that combined a questionnaire with hospital data, asking e-scooter trauma patients to self-report on their accident mechanism, including helmet use and alcohol intoxication. Study characteristics, methodologies and duration are summarised in Table 3.

**Table 3:** Study characteristics summarised.

Study	Sample size	Location	Age distribution (%) ± (median)	Sex (M% F%)	Study type, e.g., survey, retrospective
Almannaa et al. (20)	439	Riyadh, Saudi Arabia	18–30—32	M— 60	Online survey
			31–45—41		
			46–60—22	F—40	
			> 60—5		
Buehler et al. (21)	Pre-launch *n* = 462	Blacksburg, VA, United States	Not found	M—59	Survey (pre-and post-launch)
	Post-launch *n* = 428			F—41	
Gibson et al. (27)	12	Christchurch, New Zealand	22–71 range	M—25	Interviews
				F—75	
Gioldasis et al. (22)	459	Paris, France	12–17—8	M—68	Face-to-face survey
			18–24—38		
			25–29—25		
			30–34—14	F—32	
			35–44—7		
			>45—8		
Gossling (30)	173 articles	Brisbane, Australia; Christchurch, New Zealand; Copenhagen, Denmark; Dallas, United States; Los Angeles, United States; Malaga, Spain; Paris, France; Stockholm, Sweden; Vienna, Austria	N/A	N/A	Local media report analysis
		Zurich, Switzerland			
Haworth et al. (28)	775 [Shared E-scooter (SES) = 686; Private E-Scooter (PES) = 89]	Brisbane, Australia	<13—0.2	*SES* M—75.8, F—24.2	Observation of behaviours
			13–17—2.4	*PES* M—77.5, F—22.5	^*^All demographics are subjective
			Adult—97.4		
Huemer et al. (29)	253	Braunschweig, Germany	Young (18–24)—23.15	M—61.3	Observation of behaviours
			Middle-aged (25–64)—63.71	F—38.1	^*^All demographics are subjective
			Older (65+)—13.14		
James et al. (31)	181 (survey) 606 parked e-scooter	Rosslyn, VA, United States	Not given	Not given	Mixed methods—survey and observation of e-scooters
Mehdizadeh et al. (23)	395	Trondheim, Norway	14–24—27	M—42.7	Cross-sectional survey (shopping malls and snowballing)
			25–39—19	F—56.3	
			40–59—26	Other—0.0025 (1)	
			60+—28	No response—0.005 (2)	
			*Min age* 14, *max age* 98 (*mean* 43.82)		
Nikiforiadis et al. (24)	578 (271 riders; 3,017 non-riders)	Thessaloniki, Greece	*Riders*	*Riders*	Survey
			18–27—73.4	M—68.6%	
				F—31.4%	
			*Non-Riders*	*Non-riders*	
				M—45.9%	
			18–27—50.8	F—54.1%	
Sucha et al. (25)	3,385 (593; 219; 298; 491; 259)	Australia; Belgium; Czech Republic; Norway; Sweden	18–24—33; 0.46; 21, 8.8; 1.9	M—34; 62; 38; 46; 37	Online survey
			25–34—27; 11; 39; 30; 12	F—33; 36; 59; 53; 30	
			35–44—17; 15; 21; 30; 23		
			45–54—14; 21; 12; 19; 25	Other—1; 0; 0.67; 0.61; 0	
			55–64—9.3; 29; 6.4; 10; 20		
			65+—0; 24; 0.67; 2.6; 14	No answer—32; 2.3; 1.7; 0.81; 33	
			No answer—0; 0; 0; 0; 4.6		
Uluk et al. (26)	248 (120 of these also completed questionnaire)	Berlin, Germany	<18—4	Hospital data	Prospective hospital data and voluntary questionnaire
			18–25—29	M—52	
			26–40—45		
			41–64—19	F—48	
			65+—3		
Zube et al. (32)	63 (*Alcohol cohort* 57; *sober cohort* 6)	Dusseldorf, Germany	*Alcohol*	*Alcohol*	Controlled riding course observation and survey
			18–49 (29 median)	M—50.9	
				F—49.1	
			*Sober*	*Sober*	
			22–31 (26 median)	M—50	
				F—50	

No study analysed in this systematic review used United Kingdom data or opinions. Three studies were completed in Germany (26, 29, 32). Two studies used data from multiple continents, including North America, Europe, and Australasia to draw comparisons and conclusions (25, 30). The majority were single city studies.

Sample sizes ranged from 12 (27) to 3,385 (25). Due to the studies’ heterogeneity in reporting, no total sample size could be meaningfully calculated. Comparison studies between bicycles and e-scooters tended to be much larger and were generally observational (28, 29). The multinational study completed by Sucha et al. (25) also provided a large population (*n* = 3,385). The small sample size in Gibson et al. (27) (*n* = 12) was likely due to the interview style format and the timeframe of the research.

#### 3.1.3. Appraisal of studies and risk of bias assessment

**Figure 2:**
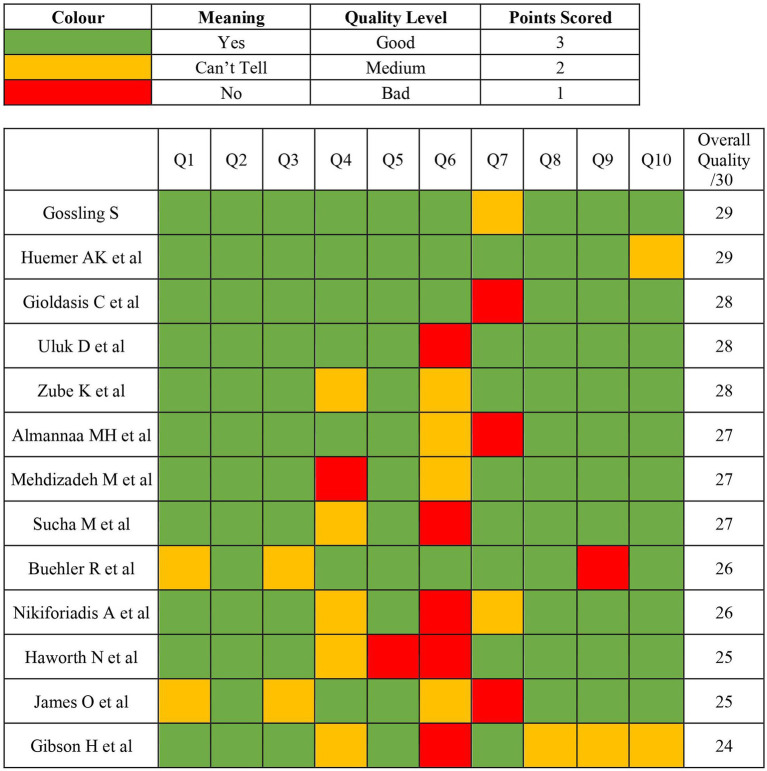
CASP analysis of included studies with value key.

The overall quality of the studies were judged as good with scores ranging from 24 to 29, with the two reviewers (NB and ZA) agreeing that a score of 24 or more was of good quality. Multiple studies had issues with the lack of clarity surrounding both the researchers’ own bias on the subject and the recruitment strategy. The quality assessment of the included studies is summarised in Figure 2.

#### 3.1.4. Thematic analysis

Multiple themes emerged from the literature: safety concerns; risk-taking behaviours of e-scooter riders; non-rider perceptions; and infrastructure. Five studies considered the motivation for riding which included: travel to public transport (20, 21, 31), speed (21, 30), for leisure (21, 28) and convenience (30).

##### 3.1.4.1. Safety concerns

A common theme across the literature was safety concerns expressed by the public and the researchers. In most of the studies (8/13), the objective was to seek out whether perceived safety concerns were felt widely (20, 22–24, 27, 28, 30, 31). Use of news articles of Gossling (30) showed the wide scope of these concerns throughout the nine countries analysed. One study questioned a discrete population pre-and post-implementation of e-scooters, remarking on the little change in concerns between the responses (21). Buehler et al. (21) concluded that concerns about the potential injury to riders and harm to other pavement or road users was still high, despite reassurances from the micromobility companies. As Haworth et al. (28) noted, there were higher levels of concern regarding safety and risk-taking behaviour of private e-scooter users compared to those using other forms of micromobility observed in their study. Two studies emphasised a need for clear rules and regulations (22, 30), whilst five studies found poor knowledge and enforcement of the existing e-scooter rules and regulations (22–24, 29, 31).

##### 3.1.4.2. Risk-taking behaviours

Risk-taking behaviours were explicitly analysed by 10/13 studies (22–30, 32). Other studies discussed secondary task behaviours that could be classified as risk-taking, such as mobile phone use, which was covered in two studies (22, 29). Gioldasis et al. (22) and Huemer et al. (29) both discussed that risk-taking behaviour was more likely with young, male riders. There was a general consensus regarding the negative effect of alcohol on riding safety (22, 23, 26, 30, 32), however, only two studies reported data on illicit drug use with e-scooter riding (22, 26). Alcohol usage was covered closely by Mehdizadeh et al. (23), who found a higher perceived acceptable blood alcohol content (BAC) than considered safe to ride. It is unclear from the literature whether these behaviours are the same in the United Kingdom, since no United Kingdom-wide studies were identified. Zube et al. (32) concluded that a BAC below 1.10 g/kg still impaired riding performance, indicating the alcohol-related risk potential whilst riding.

##### 3.1.4.3. Non-rider perceptions

Non-rider perceptions of e-scooters were considered by 6/13 studies, who reviewed this subject using various methodologies (20, 21, 24, 25, 27, 31). Within non-rider perceptions, the views on e-scooters varied. Poor parking of e-scooters and lack of infrastructure were recurring themes within some of the literature that examined non-rider perceptions and/or researcher observations (21, 24, 25, 30–32). Depending on the country, e-scooters could legally be ridden in different places, such as: pavements in Brisbane, Australia; vs. only on roads and bicycle lanes in Paris, France. When e-scooters could be ridden in multiple places, this seemed to increase confusion (31).

##### 3.1.4.4. Infrastructure

Several studies made recommendations about the creation of appropriate and safe spaces for e-scooter use (21, 22, 24, 30). Further research into whether widened bicycle lanes and pavements were suitable was encouraged by two studies (28, 31). Sucha et al. (25) added that ‘uneven surfaces’ were the most common causes of accidents in their cohort, emphasising the need for well-maintained infrastructure. Infrastructure was generally considered a secondary rather than a primary outcome.

### 3.2. Survey results

The second part of this study aimed to begin to fill these gaps in the literature by performing surveys in the West Midlands including Birmingham, the United Kingdom’s second largest city.

Due to the location of the questionnaires and the distribution methods, it was likely that the demographics were going to be heavily skewed towards the student population encircling the QEHB. This was reflected in the demographics collected from the respondents, with a significant majority in both questionnaires being 18–25 years of age. However, this age group were also probably most likely to use e-scooters and hence be relevant to this study. Multiple respondents used the free-text function to express concerns over safety as well as the lack of legislation around e-scooters. The sample sizes obtained were not statistically representative of the West Midlands population and therefore statistical analytics were not completed (Table 4).

**Table 4:** Demographics of questionnaire respondents.

		Public opinion	Road user attitudes
Sex	Male	28.7%	28.5%
	Female	69.3%	70.1%
	Prefer not to say	2.0%	1.4%
Age (years)	17 and under	0.0%	0.0%
	18–25	79.1%	81.9%
	26–40	12.4%	12.5%
	41–64	7.8%	4.2%
	65+	0.7%	1.4%
	Prefer not to say	0.0%	0.0%
Do you have a driving licence (provisional or full)	Yes	96.1%	96.6%
	No	3.9%	3.4%
E-scooter usage	Voi or other rideshare	52.9%	53.5%
	No	47.1%	46.5%

#### 3.2.1. Survey 1: the public opinion of e-scooter usage in the West Midlands

153 respondents submitted responses on the public opinion of e-scooters. 47.1% of those responding were non-riders. Almost all had a driving licence (96.1%) with a slight majority having ridden some type of rental e-scooter before (52.9%).

Opinions on the safety of e-scooters were mixed. The majority (59.5%) considered that e-scooters were unsafe whilst 15.7% were unable to agree or disagree with the statement. People raised concerns regarding the lack of clarity of e-scooter trial rules; dangerous riding behaviours, such as no helmet or switching between road and pavement; and poor parking of e-scooters, creating trip hazards. 73.8% considered that abandoned e-scooters were a danger to other footpath, cycle lane and/or road users.

An overwhelming majority reported that they did not wear a helmet when riding an e-scooter (90.1%). Over half had either ridden an e-scooter with a passenger (18.5%), as a passenger (19.8%), or both (13.6%). Around one quarter (25.9%) admitted to using an e-scooter whilst under the influence of drugs or alcohol. 11 injuries were reported (7.2%) with 9/11 managing them only at home.

Respondents’ observations and experiences of e-scooters revealed that they were ridden in multiple locations, including where they were not permitted. Road use was the highest in both observed and self-reported behaviour with designated cycle lanes also being a location of high use. Behaviour of riders observed by respondents included a high incidence of illegal footpath riding with others seen in pedestrian only zones, and across grass areas (Table 5).

**Table 5:** Rider and non-rider observations and self-reported e-scooter riding locations.

	Legal			Illegal	
	*Road*	*Cycle Lane*	*Multi-Use Path*	*Footpath*	*Other*
Reported User Behaviour (*n* = 72)	86.4%	65.4%	50.6%	50.6%	N/A
Observed Behaviour (*n* = 153)	98.0%	89.5%	84.3%	98.7%	2.6%

There were positive free-text comments on the usefulness of e-scooters as a form of micromobility, low cost, and ‘first/last mile’ distances (the first/last mile of a journey, generally walking). Rental e-scooters were praised for their indicators and visibility at night, as well as the speed limiter function. There was apprehension about children using them due to lack of road safety awareness, personal protective equipment (PPE), and dangerous riding on e-scooters observed by respondents. Respondents emphasised a need for education around safe road behaviours and improved infrastructure (14/32 {43.8%}).

#### 3.2.2. Survey 2: road user attitudes to e-scooter usage in the West Midlands

146 respondents completed this questionnaire with a slight majority having previously ridden an e-scooter (53.5%). Most respondents used a car (*n* = 114) and/or public transport (*n* = 90) as their most common modes of transport on the road.

Opinions were mixed on e-scooters as a safe mode of transport. Overall, the majority believed that they were unsafe (*n* = 87). Six people were involved in an accident involving an e-scooter as a non-rider. This confirmed that other road users are put at risk by e-scooters. Road users (7/29 free-text respondents) also commented on having to perform emergency manoeuvres to avoid e-scooters ridden dangerously or whose riders were not wearing hi-vis PPE.

An overwhelming majority believed that there was insufficient legislation or guidance available around e-scooter safety (72.4%) and 20.0% were unable to give an opinion. Respondents commented that guidance was not easily accessible, even if it existed.

Many respondents suggested unprompted that infrastructure should be adjusted nationwide to accommodate bicycles, e-scooters, and other modes of micromobility. They believed that with this added infrastructure the number of incidents could be dramatically reduced.

## 4. Discussion

Whilst perceptions, attitudes and risk-taking behaviours have been analysed outside the United Kingdom, we identified no independent research in United Kingdom literature on these topics. The quality of the literature outside the United Kingdom was good, with strong themes emerging around safety concerns, risk-taking behaviour and non-rider perceptions, amongst others. The recommendations made could assist in adjusting the structure of e-scooter trials and usage in the United Kingdom, provided United Kingdom-based independent research confirms this. Overall, our research found e-scooters were considered an environmentally friendly but potentially dangerous micromobility vehicle used for short-journey distances. Opinions within the literature and the surveys confirmed various themes around rider behaviour and safety. Recommendations were commonly made concerning improved infrastructure (21, 22, 24, 25, 28, 30, 31) and greater education for e-scooter riders (24, 29, 31).

### 4.1. Safety concerns

Safety concerns were a recurring theme across this research. PACTS estimated that 75.9% of casualties were the riders themselves, with pedestrians making up 14.6% (4). Multiple studies have recently been completed investigating e-scooter injury patterns with 2021 Trauma Audit and Research Network (TARN) data analysed by Clough et al. (3). Compared to bicycle-related trauma, e-scooter trauma patients: are more likely to require major trauma centre input (60.4 vs. 46.9%); are younger (median age 35.2 vs. 50.4); have a significantly lower rate of helmet use (7.2 vs. 47%); and are significantly more likely to be under the influence of alcohol or illicit substances (25.6 vs. 7.2%). This might explain why, despite a lower number of e-scooter trauma patients (*n* = 293) compared to bicycle (*n* = 2,538), they often require a higher level of care, adding strain to healthcare systems.

The potential risks to vulnerable pedestrians who are hard of hearing, have difficulty with their vision, or with impaired mobility are high. The RNIB published recommendations for local authorities hosting e-scooter trials on how to create a safe environment for both pedestrians and riders (13). Following this, Voi, the micromobility company responsible for the first trial in the West Midlands, collaborated with the RNIB to redesign their e-scooter parking racks, with the installation of the first visually distinctive rack being in Birmingham (33). Nevertheless, our research found that abandoned e-scooters were still a potential trip hazard.

Infrastructure developments may be key to reducing trauma and improving e-scooter safety. Voi identified three road hazards that contributed to accidents: potholes, gravel, and busy junctions, confirming Sucha et al.’s findings (25, 34). They also linked unsafe road conditions such as ice and snow to accidents. With the dual footpath/cycle lanes commonplace in many United Kingdom cities, the ‘transforming’ seen by Gibson et al. (27) in New Zealand between locations could also be a risk here.

### 4.2. Risk-taking behaviours

Risk-taking behaviours continue to contribute to potentially severe injuries. Five studies recommended public information campaigns to address this (22–24, 29, 31), and five advocated for greater enforcement of the rules (22, 23, 25, 28, 30). Helmet use was found to be low in the literature as well as in our surveys’ self-reported behaviour and behaviours observed by respondents. The low helmet use reported (90.1%), increases their risk of head and facial injuries. The QEHB maxillofacial department found that, in the 34% of their trauma patients where it was recorded, none wore a helmet (35). Uluk et al. (26) also found that only 1% of their e-scooter trauma patients wore a helmet. Rental schemes usually do not provide helmets, requiring riders to bring their own in advance. Given most rides are for first/last mile or leisure (23, 28, 30, 31), it may be less likely that a helmet will be worn. 11 free-text comments suggested that helmet use should be mandatory or monitored more closely.

This research found a relatively high incidence of alcohol consumption and impairment when riding an e-scooter (23, 26). There were more instances of riding under the influence in the 18–25 category respondents of the surveys, however statistically significant conclusions cannot be drawn. Alcohol impairment whilst riding an e-scooter may only decrease with a shift in public attitudes, similar to driving under the influence of alcohol.

Finally, tandem riding, involving riding with a passenger or as a passenger, was found to contribute to injuries and increased perceived risk (26). Around one third of the first survey admitted to this.

### 4.3. Non-rider perceptions

Non-rider perception analysis allows researchers and governing bodies to better understand the impact of implementing e-scooters. Without this, the strength of any conclusion is potentially reduced. It is illegal to ride e-scooters on the pavement in the United Kingdom, however responses on both personal usage and observations indicated that this is commonplace. Respondents observed e-scooters riding silently on pavements, giving little warning. Gibson et al. (27) discussed the unpredictable ‘transformation’ of e-scooters between different transport categories, causing unease. Variation in e-scooter riding guidelines between areas may create confusion, as noted by James et al. (31) regarding American schemes. Moreover Useche et al. (36) emphasised that road users’ lack of experience interacting with e-scooters could be a contributing factor to the uncertainty and trepidation that non-riders are currently facing.

### 4.4. Private vs. rental

A difficulty faced in analysing e-scooter perceptions and attitudes is the presence of privately owned e-scooters. Given PACTS estimates that private e-scooters account for at least 69% of United Kingdom e-scooter casualties (14), a distinction between private and rental should be made in future research.

Three studies differentiated between private and rental e-scooters (22, 23, 28), with the latter subject to stricter safety measures, including technical specifications, speed limiters and rider checks (4). These differences may impact accident rates and injury patterns, something that should ideally be understood when new United Kingdom legislation is being considered.

The negative image portrayed by the media as discussed by Gossling (30) is something that micromobility companies are working to change. Voi’s 2023 safety report highlighted shared micromobility risks, safer routes for e-scooter riders, vehicle safety improvements, and monitoring responsible rider behaviour (34). Their Vision Zero goal of eliminating fatalities and serious injuries by 2030, along with their acknowledgement of contributing risk factors to these incidents, demonstrates their awareness of the dangers associated with e-scooters. They emphasised that cities with extensive cycle lane infrastructure, like Vienna, experience lower rates of micromobility trauma compared to cities with fewer cycle lanes. Voi and Beryl, the micromobility company now overseeing the West Midlands rental e-scooters, have geofencing technology which limits e-scooter speeds to 3mph in ‘go slow zones’ (37). The rationale is to minimise collision forces in densely pedestrianised areas.

Behaviour patterns differ between private and rental e-scooters. Haworth et al. (28) found that rental e-scooters were less numerous than private e-scooters during peak commuting hours, with the opposite being true during the middle of the day. They also observed that private riders engaged in fewer illegal behaviours than their rental counterparts. Conversely, multiple survey respondents reported that behaviour endangering riders and pedestrians were more often private e-scooters rather than rental. This warrants further United Kingdom research.

United Kingdom paediatric e-scooter trauma data exist despite regulations currently limiting rental e-scooter use to those over 16 years old, which is higher than most other European countries. 6/13 of the studies reviewed had paediatric data of some kind (22–24, 26, 28, 29). Research of London paediatric orthopaedic referrals of Morgan et al. (6) in 2020 uncovered 10 patients, coded as e-scooter riders, with a median age of 15 and range of 13–17. Notably, private e-scooters have no age limit on them (4), potentially contributing to these paediatric traumas. Further examination of TARN and other United Kingdom datasets to review paediatric e-scooter trauma should be undertaken.

### 4.5. Limitations

The limitations with the systematic review were mainly around the fact the studies were observational, with little quantitative data analysed. The exclusion of non-English language studies also limits the analysis. The exclusion of MEDLINE and Scopus databases may have limited the potential literature that could have been retrieved, however, in general most studies are captured by the three databases we searched. With any systematic literature review there is also the potential for publication and selection biases to occur, which are difficult to avoid but should be acknowledged. Future studies using randomised controlled trials assessing e-scooter safety and behaviours would be of use, both in the United Kingdom and elsewhere. Once sufficient quantitative data is available on this subject, a future systematic review should be undertaken.

Limitations with our research study include the survey response rate, research timeframe and an inability to draw strong conclusions that represent the opinions of the West Midlands or United Kingdom population. Comparisons could be made if more areas were investigated using similar qualitative research methods. The large proportion of respondents being female is not representative of the Birmingham population and most e-scooter riders are male (20, 21, 24, 25, 28, 29). Furthermore, many respondents were in the age group that most commonly used e-scooters, potentially adding a bias. The use of questionnaires adds a potential social desirability bias. No distinction was explicitly made in the surveys and some of the literature between private and rental e-scooters.

A limited amount of grey literature was reviewed, which could have been expanded and statistically analysed. This was mainly found through seeking out the publications of parties who had supplied information or raised concerns to the United Kingdom Parliamentary Transport Committee on E-Scooters.

## 5. Conclusion

Our systematic literature review synthesised good quality international qualitative research on e-scooter attitudes, perceptions, and risk-taking behaviours. Alongside the quantitative studies that are emerging in the United Kingdom, our research has enabled an insight into how the West Midlands population perceives e-scooters and the factors that could be driving the rise in hospital attendances related to e-scooters. The qualitative nature of this research means that the findings may not be representative of the entire United Kingdom population, however they can be used to shape our understanding, subsequent research, and potential responses.

Perceived as a useful method of first/last mile travel, convenient and sustainable, the public opinion of e-scooters’ usefulness was positive. However, concerns remain as to their safety and the behaviour of the riders endangering not only themselves but also other road users and pedestrians.

Our research points to behaviours driving United Kingdom trauma admissions, with infrastructure improvements, rider education, and clear rule enforcement highlighted as potential preventative measures. The high number of private compared to rental e-scooters in the United Kingdom has shaped the perceptions of the public and those who use them, and future research should differentiate them before analysis.

### What is already known on this subject

Global e-scooter usage has risen dramatically with a corresponding increase in trauma. Concerns have been raised about risk-taking behaviours and the safety of riders and others. Multiple studies have been published around injury patterns and demographics of e-scooter trauma patients.

### What this study adds

The first independent review of risk-taking behaviours and attitudes surrounding e-scooters in the United Kingdom, drawing comparisons with international literature.

### How this study might affect research, practise, or policy

Future differentiation between private and rental e-scooters in hospital, police, and other datasets. Public policy changes regarding easily accessible public guidance and education for riders and non-riders of e-scooters; and improving micromobility infrastructure.

## Data availability statement

The raw data supporting the conclusions of this article will be made available by the authors, without undue reservation.

## Author contributions

NB: Conceptualization, Formal analysis, Investigation, Methodology, Writing – original draft, Writing – review & editing, Data curation. ZA: Methodology, Supervision, Writing – review & editing, Conceptualization, Validation.
