# Supplementary material for: E-scooter attitudes and risk-taking behaviours: an international systematic literature review and survey responses in the West Midlands, United Kingdom
Source: Front Public Health. 2023 Oct 13;11:1277378. doi: 10.3389/fpubh.2023.1277378 (PMC10613103; doi:10.3389/fpubh.2023.1277378)
Supplement: Supplementary file 1 [file Data_Sheet_1.docx]

**Appendix 1: E-Scooter Survey Questions**

**Road User Attitudes to E-Scooter Usage in the West Midlands**

*Preface:* Public use electric scooters (also known as e-scooters) were introduced to the West Midlands in 2020 as a new form of micro mobility. Since then, there has been little research into the opinion of road users outside of the government and e-scooter companies themselves. Birmingham City Council has completed some research into this, however this was early on in the trial period. As of 28th February 2023, the public trial in the West Midlands ended. This questionnaire aims to capture the opinions held by other users of the road. By completing this questionnaire you are consenting to your anonymous data being used for research purposes as part of the Trauma Science Masters at the University of Birmingham.

| **No.** | **Question** | **Potential Responses** |
| --- | --- | --- |
| *1* | What is your sex? | Male; Female; Prefer not to say |
| *2* | How old are you? | 17 and under; 18-25; 26-40; 41-64; 65 and over; Prefer not to say |
| *3* | Do you have a driving licence (provisional or full)? | Yes; No |
| *4* | Have you ever used a Voi e-scooter or other rental e-scooter? | Yes; No |
| *5* | Which modes of transport do you use most commonly on the road? | Car; Motorbike; Bicycle; E-scooter; Public transport – bus, tram, taxi; N/A I walk only |
| *6* | Have you been involved in an accident involving an e-scooter as a road user? (NB: not as the rider of the e-scooter) | Yes; No |
| *7* | Please select the option regarding the following statement that you most agree with: E-scooters are a safe mode of transport. | Strongly agree; Slightly agree; Neither agree nor disagree; Slightly disagree; Strongly disagree |
| *8* | Do you believe that there is sufficient legislation/guidance around safety whilst using an e-scooter? | Yes; No; Not sure/no opinion |
| *9* | Please add any further comments here. | *FREE TEXT RESPONSE* |

**The Public Opinion of E-Scooter Usage in the West Midlands**

*Preface:* Since the introduction of public hire E-scooters in the West Midlands in 2020, there has been little research into the public's opinion outside of the local councils and e-scooter companies themselves. As usage increases, it is important to gain a better understanding of the public's opinion including users and non-users. This questionnaire seeks to uncover the opinion of the public to aid future decision making and changes to legislation, including healthcare service management. By completing this questionnaire you are consenting to your anonymous data being used for research purposes as part of the Trauma Science Masters at the University of Birmingham.

| **No.** | **Question** | **Potential Responses** |
| --- | --- | --- |
| *1* | What is your sex? | Male; Female; Prefer not to say |
| *2* | How old are you? | 17 and under; 18-25; 26-40; 41-64; 65 and over; Prefer not to say |
| *3* | Do you have a driving licence (provisional or full)? | Yes; No |
| *4* | Have you ever used a Voi e-scooter or other rental e-scooter? | Yes, Voi; Yes, other; No |
| *5* | Have you ever ridden an e-scooter with a passenger or as a passenger? | Yes, with a passenger; Yes, as a passenger; No |
| *6* | Whilst riding an e-scooter, do you wear a helmet? | Yes, all or most of the time; Yes, sometimes; No, never |
| *7* | Have you ever been under the influence of drugs or alcohol whilst using an e-scooter? | Yes; No |
| *8* | Have you ever been injured whilst using an e-scooter or by an e-scooter? | Yes; No |
| *9* | Did you manage these injuries at home, for example using first aid measures? | Yes; No; N/A |
| *10* | If no to the above question, did you require other medical attention e.g. at a GP surgery and/or A&E? | GP surgery; A&E; Both; N/A |
| *11* | Please select the option regarding the following statement that you most agree with: Abandoned e-scooters are a danger to other footpath, cycle lane, and/or road users | Strongly agree; Agree; Neither agree nor disagree; Disagree; Strongly disagree |
| *12* | Where have you ridden an e-scooter? | On a footpath; In a designated cycle lane; On a multi-use path; On the road; I have not ridden an e-scooter |
| *13* | Where have you witnessed others riding an e-scooter? | On a footpath; In a designated cycle lane; On a multi-use path; On the road |
| *14* | Please select the option regarding the following statement that you most agree with: E-scooters are a safe mode of transport. | Strongly agree; Slightly agree; Neither agree nor disagree; Slightly disagree; Strongly disagree |
| *15* | Please add any further comments here. | *FREE TEXT RESPONSE* |
